# Epidemiology of Dog and Cat Abandonment in Spain (2008–2013)

**DOI:** 10.3390/ani5020364

**Published:** 2015-06-12

**Authors:** Jaume Fatjó, Jonathan Bowen, Elena García, Paula Calvo, Silvia Rueda, Silvia Amblás, Jaume F. Lalanza

**Affiliations:** 1 Chair Affinity Foundation Animals and Health, Department of Psychiatry and Forensic Medicine, School of Medicine, Universitat Autònoma de Barcelona, 08193 Bellaterra, Barcelona, Spain; E-Mails: jbowen@rvc.ac.uk (J.B.); etovet.elena@gmail.com (E.G.); paula.calvo@e-campus.uab.cat (P.C.); jaumeferr@gmail.com (J.F.L.); 2 Queen Mother Hospital for Small Animals, The Royal Veterinary College, Hawkshead, North Mymms, Hatfield, Herts AL9 7TA, UK; 3 DEP Institute, C/Aragó 631-633, Local 1-2, 08026 Barcelona, Spain; E-Mails: srueda@dep.net (S.R.); amblas@dep.net (S.A.); 4 IMIM (Hospital del Mar Medical Research Institute), c/Doctor Aiguader 88, 08003 Barcelona, Spain

**Keywords:** dogs, cats, abandonment, animal shelter, identification

## Abstract

**Simple Summary:**

In this paper we wanted to estimate the incidence of abandonment, as well as the general profile of dogs and cats entering animal shelters in our country. Also, we wanted to test the impact of identification on the recovery of dogs that had entered animal shelters. More than 100,000 dogs and more than 30,000 cats enter animal shelters annually in Spain. We observed a seasonal effect in the number of admissions in cats. A considerable percentage remained at the shelter or was euthanized. We found that identification of dogs with a microchip increased by 3-fold the likelihood of them being returned to the owner.

**Abstract:**

Millions of pets are abandoned worldwide every year, which is an important animal welfare and financial problem. This paper was divided into three studies. Our first two studies were designed as a national survey of animal shelters to profile the population of stray dogs and cats, as well as to gather information on both relinquishment and adoption. The aim of our third study was to test the impact of identification on the recovery of dogs entering animal shelters. Studies one and two indicate that more than 100,000 dogs and more than 30,000 cats enter animal shelters annually in Spain. We observed a seasonal effect in the number of admissions in cats. Two-thirds of dogs and cats entering shelters were found as strays, while the rest were relinquished directly to the shelter. Most pets admitted to animal shelters were adult, non-purebred, and without a microchip, with the majority of dogs being medium sized. Adult dogs spent significantly more time in shelters than puppies. While most animals were either adopted or recovered by their owner, a considerable percentage remained at the shelter or was euthanized. The identification of dogs with a microchip increased by 3-fold the likelihood of them being returned to the owner.

## 1. Introduction

Pets tend to be viewed as family members; they are an important source of affection and attachment and are an integral part of our society [1]. However, millions of dogs and cats are abandoned worldwide every year, which is an important issue because, first, it is considered a major animal welfare problem [2], and second, the care of stray pets carries a large public and private cost (e.g. $2 billion per year in the in the US [3]). From this economic viewpoint, data based on representative national surveys are crucial for developing private and public policies to reduce pet abandonment and promote adoption. Also, gaining an understanding of the profile and needs of abandoned animals is the first step toward improving their welfare and quality of life. 

To make progress in this area, we need accurate figures for the rate of abandonment and the rate of rehoming of cats and dogs that enter a shelter each year. To calculate the rate of abandonment we need an estimate of the total population of dogs and cats and the number abandoned, but there are no consistent official data on stray animals in western countries, and most available data comes from animal associations and non-profit shelters. For example, the American Society for the Prevention of Cruelty to Animals estimates that 5–7 million pets are admitted to shelters each year in the US [4]. In the UK, an estimated 126,100 and 110,000 stray dogs were handled by local councils in 2011 and 2014, respectively [5]; and 246,397 stray dogs and cats in 2010 [6]. There are no official data in Spain either on the number of animals abandoned every year, or on the total population of dogs and cats. It is also necessary to distinguish between truly abandoned animals, and those that are simply lost and could be eventually recovered by their owners. 

It is very important to profile the population of dogs and cats that are housed in shelters, as well as the abandoners and potential adopters, as a means of reducing the number of abandoned animals and improving the number and quality of adoptions. Although there is a dearth of peer-reviewed evidence and official data, dogs admitted to shelters are reported to be typically adult, small, male and sexually intact [7,8]. There is even less data for cats [9]. There is also limited population-level data about the profile of abandoners and potential adopters. The main reported reasons for relinquishment are accommodation problems (including owner relocation), behavioral issues in the dog, and the amount of work/effort/time required to take care of the dog [7]. Similarly, the main reasons for returning dogs to the shelter after failed adoption are behavioral problems, the amount of commitment required, and landlord problems [10].

Microchip identification is considered one of the main strategies to prevent dog abandonment and to facilitate the recovery of lost dogs. The identification of dogs and cats in Spain is compulsory in some autonomic regions, but not in others. To our knowledge no studies have measured the impact of microchipping on the recovery rate of lost dogs that enter animal shelters.

The specific objectives of our research are: (i) to quantify the population of domestic dogs and cats in Spain as a first step to understand the abandonment problem; (ii) to describe the magnitude of the animal abandonment problem in Spain; (iii) to estimate the real number of abandoned dogs and cats as opposed to those that got lost; (iv) to explore the main reasons for relinquishment and the motivations for adoption; (v) to evaluate the differences between dogs and cats, particularly in terms of their final destination after being admitted in a shelter; and (vi) to test the impact of identification on rehoming rate.

To achieve these objectives, we carried out three different studies: (1) a population-based survey to estimate the total population of domestic dogs and cats in Spain between 2008 and 2013; (2) a survey of animal-shelters to estimate the number of stray dogs and cats and their main characteristics as well as the overall profile of the abandoners and the adopters during the same period (2008–2013); (3) a study in 30 cities around the city of Barcelona to test the impact of identification in the abandonment-recovery process in dogs. 

## 2. Experimental Section 

### 2.1. Study 1

Data were collected from a population-based sample of 1,045 Spanish subjects and repeated annually from 2008 to 2013. Samples were randomly selected from the database of the Spanish National Statistical Institute (INE) with a confidence level of 95.5% and a 3% standard error. The sample population included people older than 15 years old from municipalities with more than 2000 inhabitants. The sample was proportional to the population of the nine main geographical areas in Spain and included 23% of participants from municipalities with less than 10,000 inhabitants, 30% from municipalities between 10,000 and 100,000 inhabitants, 21% from municipalities between 100,000 and 750,000 inhabitants and 26% from large metropolitan areas with more than 750,000 inhabitants. The sampling method also took into account the following socio-demographic factors: gender, age and socioeconomic status.

Data were obtained by phone, following a standard CATI methodology (Computer-assisted telephone interviewing) [11]. In 2013, data on identification by microchip was added for both dogs and cats. Questions included general demographic information and a variety of questions on pet ownership. For the purpose of the present study, the most relevant information was the proportion of the Spanish population owning a dog or a cat.

### 2.2. Study 2

A survey of public and private animal shelters in Spain was carried out between 2008 and 2013. Due to the lack of official data, to develop an accurate census of animal shelters, we followed 3 parallel and complementary routes. First, we contacted local authorities of municipalities with more than than 10,000 inhabitants. Under Spanish law, local authorities of towns with more than 10,000 inhabitants have full responsibility for dog abandonment issues. Second, we contacted national and regional associations of private shelters, particularly to check for small private shelters that could go unnoticed by the authorities. In any case, only institutions legally established either as foundations or as non-profit organizations were included in our database. Third, we consulted the Affinity Foundation database of public and private shelters in Spain. As a result of this procedure, our database has increased over the course of the study. We included 289 non-profit organizations and private shelters in 2008, 296 in 2009, 307 in 2010, 315 in 2012 and 356 in 2013. We included 742 councils in 2008, 751 in 2009, 752 in 2010, 781 in 2012 and 778 in 2013. The participation rates of invited associations and councils were 91.9% and 82.6% respectively. 

Information about the number and general characteristics of the animals, the owners who relinquished their pets to the shelter and the adopters was obtained though a single self-reported questionnaire (in a checklist format) completed by the director of each animal shelter. Questions included: number of total admissions (annual and for every four-month period in order to explore seasonal variations), number of adoptions (total and for every four-month period), average duration of stay in the shelter, final destination of the animals, reasons to relinquish, reasons to adopt, as well as general characteristics of the animal, such as age, size classification, breed and identification by microchip. Animals staying in the shelter for more than one year were included in the category “still in shelter”. For years 2010, 2012 and 2013 information was also available on the percentage of admitted animals in shelters being strays and those being directly relinquished by their owners. For the purpose of this paper we considered the following definitions:

- Stray animals: non-owned animals, either because they are lost or abandoned and not being under the direct control of an animal shelter.
- Abandoned animals: dogs and cats purposely left by their owners, but not taken directly to the shelter.
- Relinquished animals: dogs and cats taken to the shelter by their owners.
- Admitted animals: dogs and cats entering a shelter because they have been relinquished or found as strays.

### 2.3. Study 3

Data from January to December (2009) were obtained directly from the database of SPAM, a non-profit organization providing shelter services to 30 cities around Barcelona. For reasons of confidentiality, requested information was provided by the organization following the author’s instructions. The total number of admissions and whether the dogs were relinquished to the shelter by their owners, were found as strays, or were legally held animals were recorded. Information on sex, breed, size, age and identification status was obtained for all admitted dogs. The number of dogs successfully returned to the owner and the main reasons for failure to return dogs with microchips were also recorded.

### 2.4. Statistical Analysis

Paired comparisons were carried out using the non-parametric Mann-Whitney *U* (GraphPad Prism 5) and Chi-Square (SPSS, version 18.0). For all comparisons *p* < 0.05 was considered to be significant.

## 3. Results

### 3.1. Study 1: Estimated Number of Domestic Dogs and Cats between 2008 and 2013

**Figure 1:**
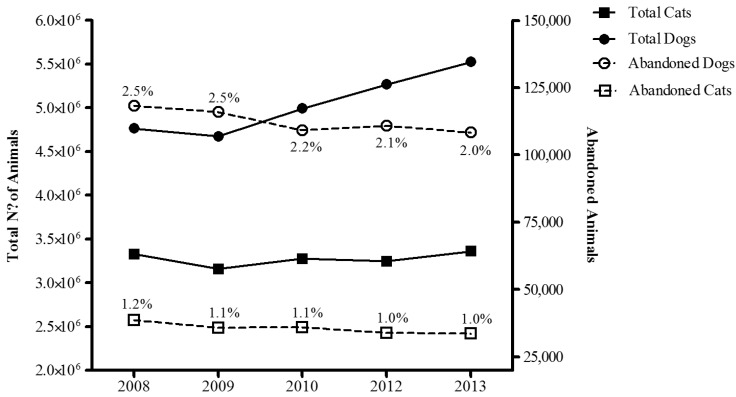
Total number of dogs (black circles - solid line) and cats (black squares- solid line) (left axis) in Spain and the estimated number of dogs (white circle - broken line) and cats (white squares – broken line) entering animal shelters in Spain from 2008 to 2013. The percentage of animals from the total population of dogs and cats entering animal shelters is also indicated entering animal shelters.

Figure 1 (left axis) shows the total number of domestic dogs and cats in Spain between 2008 and 2013. There were 4,764,000 dogs in 2008, 4,674,000 in 2009, 4,993,000 in 2010, 5,084,000 in 2011, 5,268,000 in 2012, and 5,524,000 in 2013, representing a 16% increase over 6 years. There were 3,328,000 cats in 2008, 3,158,000 in 2009, 3,274,000 in 2010, 3,212,000 in 2011, 3,246,000 in 2012, and 3,359,000 in 2013, representing a 1% increase between 2008 and 2013.

In 2013, 80.9% of all domestic dogs and 25.3% of all domestic cats were identified with a microchip.

### 3.2. Study 2: Abandonment and Relinquishment of Cats and Dogs

#### 3.2.1. Total Number of Dogs and Cats Taken by Animal Shelters

The number of dogs relinquished or admitted to animal shelters has decreased in Spain in the last 6 years (Figure 1, right axis): 118,227 in 2008, 115,879 in 2009, 109,074 in 2010, 110,809 in 2012, and 108,303 in 2013. There was also a decrease in the number of cats admitted to shelters: 38,631 in 2008, 35,794 in 2009, 35,983 in 2010, 33,851 in 2012, and 33,532 in 2013. Thus, while the total populations of dogs and cats both increased during the study period, the numbers of admissions decreased slightly. Note that the percentage of dogs entering shelters was twice that of abandoned cats.

Taking into consideration the official census of the Spanish human population, the estimated incidence of dog admissions in shelters per 1000 inhabitants was 2.6 in 2008, 2.5 in 2009, 2.3 in 2010, 2.3 in 2012 and 2.3 in 2013. For cats, the estimated incidence of admissions in shelters per 1000 inhabitants was 0.8 in 2008, 0.77 in 2009, 0.77 in 2010, 0.7 in 2012 and 0.7 in 2013.

**Figure 2:**
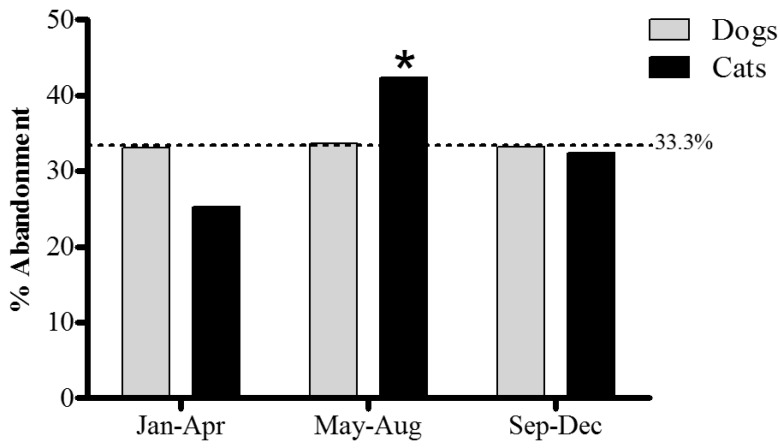
Seasonal percentage of admissions to shelters in 2013 for dogs (grey bars) and cats (black bars). First season: January (Jan) to April (Apr), second season: May to August (Aug), third season: September (Sep) to December (Dec). * *p* < 0.02 Paired Comparisons (U Mann-Whitney): second *vs.* first, third.

Time of year seems to be a factor for the rate of admission in shelters, although only in cats (Figure 2). In 2013, the admission rate in dogs was remarkably consistent throughout the year (33.1% in January–April, 33.7% in May–August and 33.2% in September–December), see Figure 2. In the same year, admission rate in cats fluctuated, with a dip in January–April (25.3%), and a peak in May–August (42.3%) (*p* < 0.02). Data are shown for 2013 only, as there were no significant changes in these seasonal patterns between 2008 and 2013.

The percentage of animals found by the shelter as strays in 2010, 2012 and 2013 were respectively 60.7%, 66.8% and 67.5%, while the rest were relinquished directly to the shelter by their owners.

#### 3.2.2. Profile of Stray Dogs and Cats

The main characteristics of stray dogs and cats are shown in Table 1 (mean values, 2008–2013). Most animals were non-purebred, with just 18.3% and 9.9% of purebred and/or pedigree dogs and cats, respectively. The largest proportion of stray dogs and cats were adult (56.4% and 50.2%, respectively), followed by puppies and kittens (27.3% and 38.9%, respectively). The smallest proportion of stray animals was older animals (16.5% and 11%, respectively). 

In terms of size, medium sized dogs were significantly more common than small or large dogs (43.8%, *p* < 0.02). Size differences were only assessed for dogs, since there is little size variation among cats. The majority of stray animals were healthy when admitted to the shelter (dogs, 68.1%; cats, 60.7%, *p* < 0.02), and while most did not have a microchip, dogs were 8 times more likely to carry one than cats (26.9% and 3.2%, respectively; *p* < 0.008). The impact of identification is explored further in Study 3 (Table 3).

**Table 1:** Description of dogs and cats admitted to shelters from 2008 to 2013.

		2008	2009	2010	2012	2013	Total (mean)
**Dogs**	**With Microchip**	23.8	25.4	31.2	27.9	26.0	26.9^##^
	**Pedigree / Breed**	19.3	15.6	18.4	19.6	18.8	18.3
	**Puppy**	28.7	28.1	26.3	28.8	24.8	27.3*^##^
	**Adult**	51.5	55.1	57.6	56.6	60.1	56.2*
	**Senior**	19.8	16.9	16.1	14.7	15.1	16.5*
	**Small Size**	30.4	29.3	27.1	30.3	26.0	28.6
	**Medium Size**	41.7	43.5	43.6	43.9	46.2	43.8*
	**Large Size**	27.9	29.3	29.3	25.8	27.8	28.0
	**Healthy**	68.5	61.5	66.5	71.8	72.3	68.1
	**Diseased**	21.6	26.6	20.4	17.1	17.1	20.6
	**Injured**	9.9	11.9	13.1	11.1	10.6	11.3
**Cats**	**With Microchip**	3.3	3.5	4.6	2.9	1.8	3.2^##^
	**Pedigree / Breed**	13.4	6.6	10.9	10.8	7.7	9.9
	**Puppy**	37.2	35.4	34.1	45.7	42.2	38.9*^##^
	**Adult**	48.1	52.2	55.8	45.8	48.9	50.2*
	**Senior**	14.7	12.4	10.1	8.7	8.9	11.0*
	**Healthy**	59.0	57.5	59.7	63.3	64.1	60.7
	**Diseased**	33.5	33.5	25.3	22.3	22.3	27.4
	**Injured**	7.5	9.0	15.0	14.4	13.6	11.9

All values are expressed as %. * <0.02 between the same type of characteristic, dogs and cats separately; ## <0.008 Paired Comparisons (U Mann-Whitney): dogs *vs.* cats. n = 111–160 (shelters).

#### 3.2.3. Features of Abandonment and Adoption 

**Figure 3:**
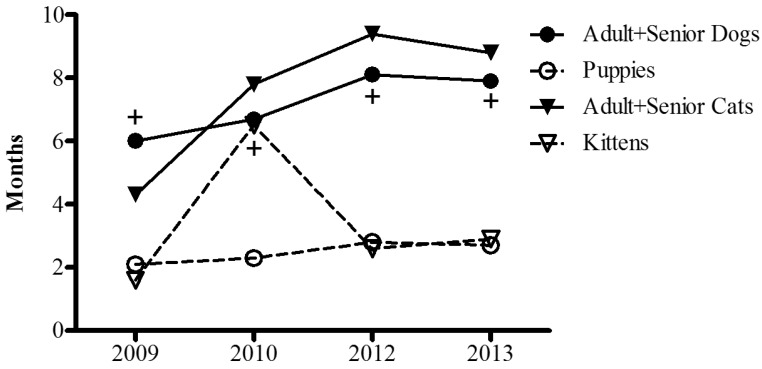
Time (in months) spent in the shelter in before being adopted, recovered or euthanized from 2009 to 2013. Black circles and continuous lines correspond to adult/senior dogs, white circles and broken lines to puppies, black triangles and continuous line to adult/senior cats, and white triangles and broken lines to kittens. + *p <* 0.03 Paired Comparison (mean of 4 years, U Mann-Whitney): adult/senior *vs.* puppy dogs. n = 105–137 (shelters).

Figure 3 shows the time (months) that stray dogs and cats spent in shelters, according to their age. Between 2009 and 2013, adult and older dogs stayed for a mean of 6 to 8.1 months, whereas puppies stayed between 2.1 and 2.8 months (*p* < 0.03). A similar trend was observed in cats (*p* = 0.06).

Table 2 shows the final destination of animals taken in by shelters, with nearly half of them being adopted. However, there were significant differences between dogs and cats: 17% of dogs were recovered by their owners, while only 4.2% cats were recovered (*p* < 0.008). 12.6% of dogs and 23.4% of cats were euthanized (*p* < 0.02).

**Table 2:** Destination of abandoned dogs and cats from 2008 to 2013.

		2008	2009	2010	2012	2013	Total (mean)
**Dogs**	**Adopted**	29.4	39.2	44.8	50.9	46.9	42.3
	**Still in Shelter**	30,7	25.5	15.7	14.6	15.1	20.3
	**Euthanized**	14,7	15.5	15.6	10.7	6.3	12.6^#^
	**Recovered**	14,0	16.8	17.2	17.0	19.9	17.0^##^
	**Other**	11,1	3.0	6.7	6.8	11.8	7.9
**Cats**	**Adopted**	19,3	27.6	37.5	39.4	36.9	32.1
	**Still in Shelter**	30,6	38.2	15.0	13.5	13.2	22.1
	**Euthanized**	22,4	18.6	30.1	23.3	22.4	23.4 ^#^
	**Recovered**	4,9	4.7	4.5	2.8	4.2	4.2 ^##^
	**Other**	22,8	11.0	12.9	20.9	23.3	18.2

All values are expressed as %. # <0.05, ## <0.008 Paired Comparisons (U Mann-Whitney): dogs *vs.* cats. n = 272 (shelters).

**Figure 4:**
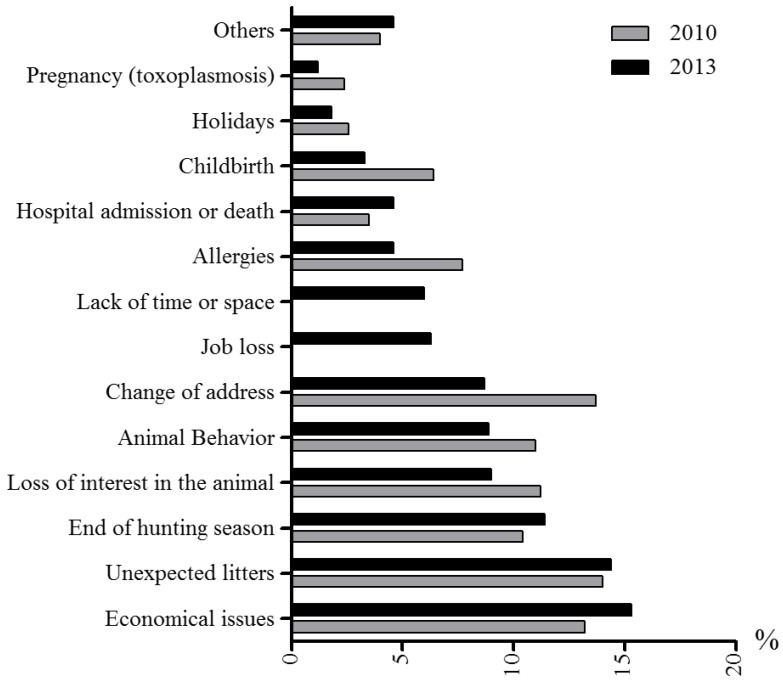
Reasons to abandon a dog or a cat in 2010 (grey columns) and in 2013 (black columns). n = 110–122 (shelters).

**Figure 5:**
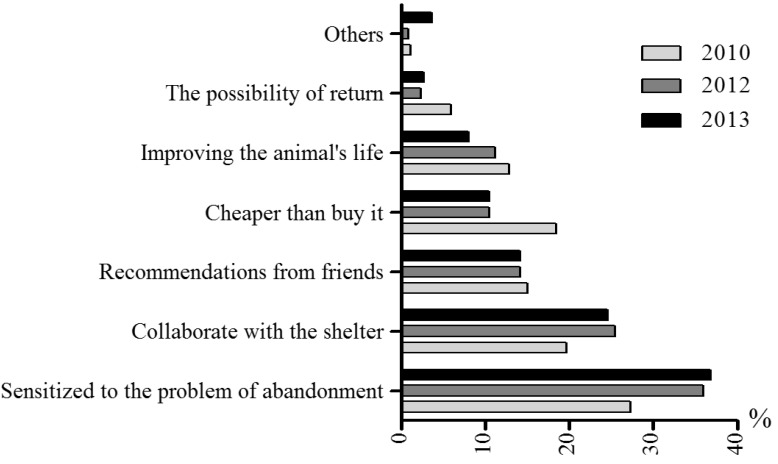
Reasons to adopt a dog or a cat in 2010 (light grey columns), in 2012 (dark grey columns) and in 2013 (black columns). n = 126–133 (shelters).

Figure 4 shows the reasons given for abandoning a dog or a cat, for the period 2010–2013. While the most common reasons in 2010 were “unexpected litters”, “change of address” and “economic issues”, this tendency changed by 2013, in which “economic issues”, “unexpected litters” and “end of hunting season” (there is an extended tradition of hunting with dogs in Spain. Some hunters abandon their dogs once the hunting season is over) were the most common reasons. Figure 5 shows the reasons for adopting: the most common reason in 2010, 2012 and 2013 was being “sensitized to the problem of abandonment”, whereas there were two notable changes in 2012 and 2013, in which “collaboration with the shelter” (*i.e.*, helping the shelter to relocate their animals) and “adopting is cheaper than buying” became more and less common, respectively. The possibility to return the dog to the shelter was also reported as a reason to adopt a pet from a shelter.

### 3.3. Study 3: The Importance of Identification: A Case Study in the Barcelona Area

A total of 1,467 admissions were recorded between 1 January and 31 December, 2009, of which 152 (10.4%) were relinquished by their owner, 1,308 (89.1%) were strays, and 7 (0.5%) were legally held dogs. Subsequent analyses include data only from those cities that have a complete stray animal surveillance service (18 out of 30). 

Complete records on sex, breed and age were available for 753 dogs. Of all admissions, 620 (82.3%) were adult dogs, 133 (17.7%) were puppies, 459 (61%) were males, 294 (39%) were females, 220 (35.4%) were large, 208 (33.5%) were middle-sized, and 192 (30.9%) were small dogs. According to the available information, 552 (73%) dogs were classified as non-purebred, and 201 (27%) as purebred.

Eight hundred and twenty dogs from 18 cities were admitted, with an annual incidence of 2 admissions per 1,000 habitants. Two hundred and seventy-two dogs (34%) were successfully returned to their owners, 544 dogs were adopted during 2009, 259 (47.6%) by people from the same city where the animal was collected. 

At the time of admission, 197 (25%) stray dogs had a microchip and 595 (75%) did not. The proportion of dogs successfully returned to the owner was significantly higher among those with a microchip (146 out of 197; 74%) than those without (126 out of 595; 21%) (Table 3: Chi-Square = 216,70, df = 1, *p <* 0,001). The main reasons for failure to return dogs with microchips included inability to contact the owner (n = 23; 45%), delays in incorporating files into the central database of identified dogs (n = 17; 33%), and refusal by the owner to take care of the animal (n = 11; 22%).

**Table 3:** The impact of identification on the rate of recovery of dogs entering animal shelters.

	Abandoned Dogs	Recovered Dogs
**With Microchip**	197 (25%)	146 (74%) ^λ^
**Without Microchip**	595 (75%)	126 (21%) ^λ^

^λ^ *p* < 0.001 (Chi-Square).

## 4. Discussion

While our results, which show a decrease in the percentage of admissions to shelters during the study period (Figure 1), could be considered positive, the percentage of admissions is still higher than before the current economic crisis. According to previous un-published surveys, in 1998, there were 94,063 stray dogs and 16,390 cats in Spain, and, respectively, 94,664 and 13,415 in 2000, 98,803 and 14,097 in 2004 [12], which is considerably fewer than in 2013 (108,303 and 33,532, respectively). The effect of the economic crisis may also be reflected in the reasons for relinquishment, with financial problems and financially-related issues like change of address and job loss being cited as the reasons for 26.9% and 30.3% of abandonments in 2010 and 2013, respectively.

Not all dogs and cats admitted to animal shelters have actually been abandoned by their owners. According to our results from studies 1 and 2, the annual incidence of admissions to shelters in the year 2009 was 2.5 per 1000 inhabitants. Excluding from the calculation the estimation of dogs that were successfully returned to their owner (16.8%), the incidence of abandonment would be reduced to 2 per 1000 inhabitants. Using a completely different methodology, data from study 3 indicates an incidence of admissions comparable to studies 1 and 2 (2.24/1000 inhabitants). Nevertheless, when dogs successfully returned to their owners are removed, the index of abandonment is reduced to 1.34/1000 inhabitants. The lack of confluence on the estimated index of abandonment between studies 1 and 2, and study 3 does not seem to be due to the percentage of dogs microchipped at the time of admission, which is almost identical in both populations (25.4% and 25% respectively). This discrepancy could be explained by methodological and geographical differences, as well as by differences in management policies between the animal shelters that took part in study 3 and the rest of animal shelters surveyed in study 2 as a group. In fact, the shelter that provided data for study 3 is particularly well known within Spain for its very efficient communication strategies. This would stress the need to promote active communication policies among animal shelters.

There were no seasonal differences in the admission rate of dogs in shelters. Contrary to popular belief, this suggests that dog abandonment is not particularly related to the summer holiday period. In fact, holiday-related issues are not reported as an important reason to relinquish dogs to animal shelters in our survey (Figure 4). 

Cats have a more seasonal reproductive cycle, usually becoming pregnant at the end of winter and giving birth in spring [13], which could explain the increase in the number of cats entering shelters between May and August. Again this interpretation is consistent with another of our results; the second most frequently reported reason for relinquishment was “unexpected litters” (Figure 4).

The increased proportion of adult animals in shelters could just reflect a higher proportion of this age category in the overall pet population. However, in our study adult dogs and cats stayed in shelters longer than puppies and kittens, which suggests an adoption bias towards the latter. Previous studies had shown that one third of adopters show a strong preference for adopting a puppy rather than an adult dog [14]. That could be explained by a general preference for adopting young individuals, but also by the social perception that adult dogs from a shelter may have behavioral problems [14,15]. However, this perception is contradicted by scientific findings. Recent research found no major correlation between puppies’ behavior and their behavior in adulthood (only in exploratory activity) [16]. In fact, it has also been reported that dogs obtained from pet stores are more likely to show aggressive behavior, fear and soiling problems than those obtained from non-commercial breeders [17]. Further, the behavior of dogs and cats is strongly influenced by the physical and the social environment, which ultimately depend on the owner’s attitudes and behavior [10,18]. Again, animal shelters, humane societies and science should make a special effort to educate people on normal canine behavior and to show the potential benefits of adopting an adult or even a senior pet.

Regarding the percentage of pets entering animal shelters identified by microchip in 2009, data from study 3 (25%) are consistent again with the results of study 2 in the same geographic area (26.9%). From study 3, we are able to conclude that identification with a microchip provides a 3-fold increase in the likelihood of a dog being returned to its owner (74%, Table 3). Making a projection to the entire population of dogs admitted in shelters, a 10% increase in the use of microchip identification would potentially result in the return of 8,123 more dogs to their owners. Our observation that dogs carrying microchips were significantly more likely to be successfully returned to their owner confirms the value of identification as a tool to reduce admissions to animal shelters [19]. However, it could also be a reflection of the fact that owners who are more willing to provide adequate care for their animals, including identification, are not only less likely to abandon them but also more likely to try to find them after they are lost. A quarter of microchip-tagged dogs admitted to a shelter were not successfully returned to the owner, mainly because of an apparent delay between implantation of the microchip and the availability of the information in the central database. Taken together, these results emphasize the need to carry out additional legal and educational measures to promote the proper identification of dogs and cats.

The proportion of dogs euthanized in animal shelters in Spain could be considered lower than that estimated in other countries For example, 3.7 million pets were euthanized in 2009 in USA [20] and 25.6% of stray dogs were euthanized in Australia in 2012 [21]. This could be related to a 2006 prohibition of euthanasia as a mean of population control in animal shelters in some regions of Spain (e.g., Catalonia).

The rate of euthanasia in cats was considerably higher than in dogs during the study period. Data from other countries also indicates that more cats than dogs are euthanized in animal shelters [21].

The possibility of being able to return the pet to the shelter after a failed adoption was reported by adopters as one of the top 5 reasons for choosing to adopt (data from 2010, 2012 and 2013). This could be related to the perception that shelter pets could be predisposed to show behavior and adaptation problems. One-third of Australian people interested in dogs consider that dogs housed in shelters have behavioral problems [14], which is also consistent with reports showing that behavioral problems are one of the most common reason for returning dogs to shelters after adoption [10,18,22,23]. Knowing that owners who receive behavioral advice are less likely to relinquish than those who do not get any support, providing behavioral advice post adoption has been suggested as a key strategy to reduce the rate of return [24]. Our results suggest that animal shelters should make efforts to inform potential adopters about the availability of post-adoption behavioral advice, including the possibility to return the pet to the shelter.

In terms of methodology, it should be remembered that we obtained data through self-reported questionnaires and that is already a limitation in our investigation. For the future we strongly believe that an official database of public and private animal shelters should be available. Also, a representative panel of shelters could be established to obtain direct data from databases, as opposed to self-reports.

**Figure 6:**
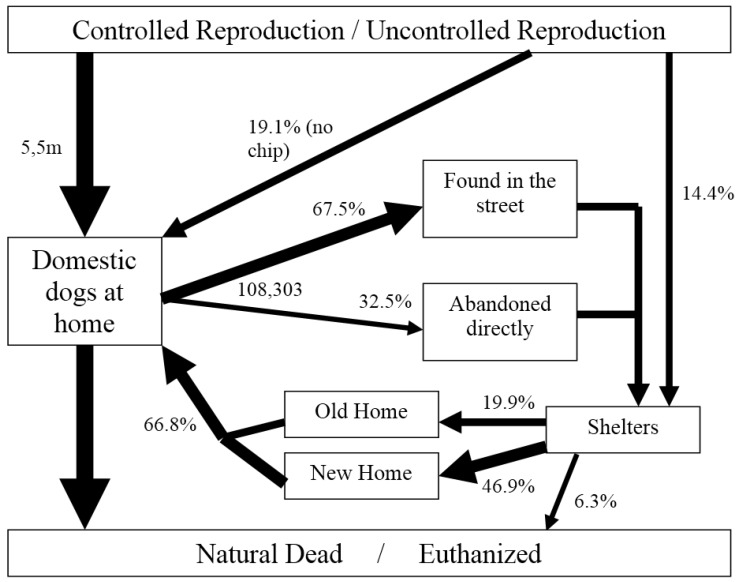
Ecology model of the domestic dog in Spain in 2013. m = millions (modified from [25]).

**Figure 7:**
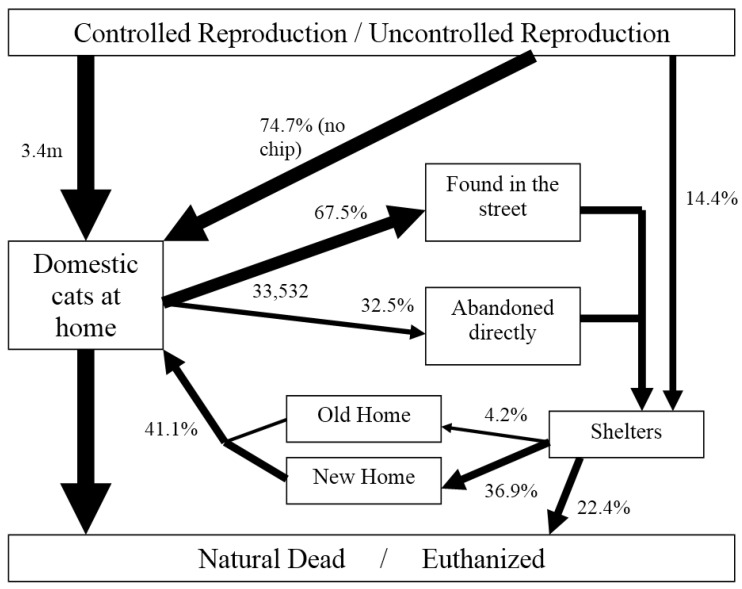
Ecology model of the domestic cat in Spain in 2013. m = millions (modified from [25]).

In summary, we have collected information to present “for the first time” not only an overall view of the problem of pet relinquishment and abandonment in Spain, but also information on the population of dogs and cats in our country. Our results for dogs and cats are summarized in Figure 6 and Figure 7 respectively, following the model presented by Miklosi to understand the demography and population dynamics of the domestic dog [25].
